# Supplementary figures and images for: BRAF V600E Detection in Liquid Biopsies from Pediatric Central Nervous System Tumors
Source: Cancers (Basel). 2019 Dec 25;12(1):66. doi: 10.3390/cancers12010066 (PMC7016762; doi:10.3390/cancers12010066)

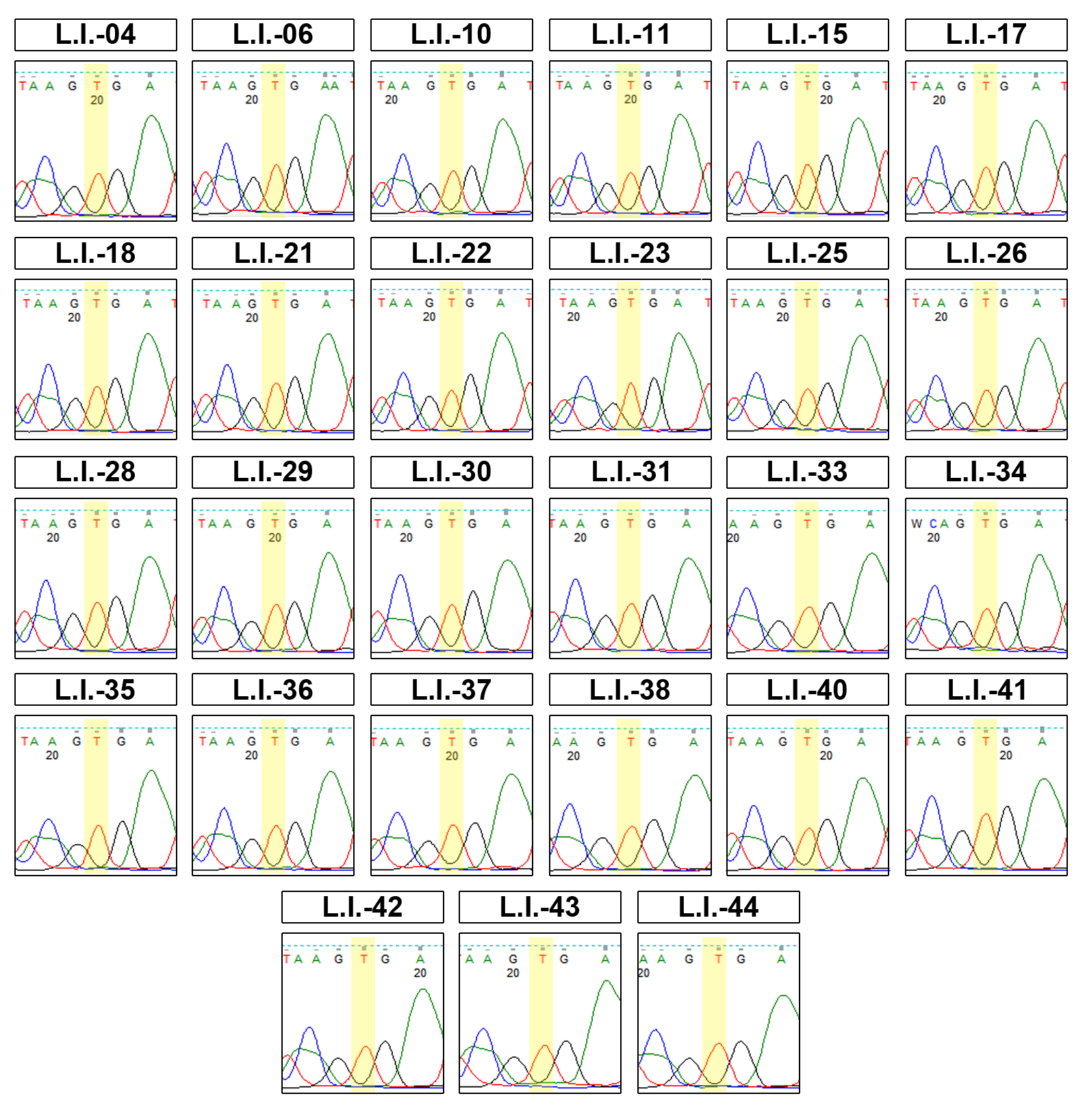

Supplement: Supplementary file 1 [file cancers-12-00066-s001.zip › Supplementary figure1.tif]
